# Effect of feeding different dietary levels of energy and protein on growth performance and immune status of Vanaraja chicken in the tropic

**DOI:** 10.14202/vetworld.2016.893-899

**Published:** 2016-08-23

**Authors:** Shahla Perween, Kaushalendra Kumar, Chandramoni, Sanjay Kumar, Pankaj Kumar Singh, Manoj Kumar, Amitava Dey

**Affiliations:** 1 Department of Animal Nutrition, Bihar Veterinary College, Bihar Agricultural University, Patna, Bihar, India; 2 Department of Veterinary Microbiology, Bihar Veterinary College, Bihar Agricultural University, Patna, Bihar, India; 3 Division of Animal Science, ICAR- Research Complex for Eastern Region, Patna, Bihar, India

**Keywords:** body weight gain, economics, energy, immunity, protein, Vanaraja

## Abstract

**Aim:**

The present study was conducted to observe the effect of feeding dietary level of energy and protein on growth performance and immune status of Vanaraja chicken in the tropic.

**Materials and Methods:**

The experiment was conducted for 56 days on 540 1-day-old chicks, which were individually weighed and distributed into nine groups having 60 birds in each. Each group was further subdivided into triplicates having 20 birds in each. Nine different experimental rations were formulated with three levels of protein, *viz*., 17%, 19%, and 21%; each with three levels of energy (2600, 2800, and 3000 kcal metabolizable energy [ME]/kg), respectively. Group T_8_ serves as control fed with 21% protein and 2800 kcal energy as per Project Directorate of Poultry, Hyderabad given requirement. Feed consumption, live weight gain, body weight change, and feed conversion ratio (FCR) were calculated based on the amount of feed consumed every week. All the birds were vaccinated following standard protocol. The hemagglutination inhibition (HI) test have been performed to assess the immunity potential of birds due to dietary effect, and serum samples were subjected to HI test at 7, 14, 21, and 28 days of age. Finally, economics of broiler production was calculated on the cost of feed per kg live weight gain.

**Results:**

This study revealed that the effect of feeding different levels of energy and protein on growth parameters such as body weight gain and FCR was found to be significantly higher (p<0.05) containing 19% and 21% crude protein with 3000 kcal ME/kg in Vanaraja birds. There was a gradual increase in antibody titer against New castle disease virus as the level of protein and energy increase. It is speculated that the better body weight gain corroborate health and antibody titer. Moreover, the better immune response recorded in the study might be due to better nutrient utilization and its extension toward the better immune response. Higher energy with medium protein diet positively reflects to obtain desirable performance economically.

**Conclusion:**

It was positive inclination toward ration containing high protein and energy which influence the immune response of Vanaraja birds to obtained desirable performance economically also.

## Introduction

In the present scenario, poultry farming is gaining strength with fast pace of development both in developed and developing countries, especially in India, the major population is dependent on agriculture and allied for their livelihood security. Currently, the total poultry population in our country is 729.21 million numbers [1], and egg production is around 74.75 billion numbers during 2013-14. The current per capita availability (2013-14) of the egg is around 61 eggs per year. The poultry meat production is estimated to be 2.69 million metric tons.

Backyard poultry farming by and large was a low input venture [2]. Besides income generation, backyard poultry farming helps in alleviation of malnutrition of the rural people through the production of valuable animal protein and empowers rural women [3,4]. In spite of low productivity, the contribution of backyard poultry toward Indian egg production is about 30-40% [5]. The backyard breed namely Vanaraja developed by the Project Directorate of Poultry (PDP), Hyderabad are very well acclimatized to village climate with good growth and moderate egg production as per the performance study conducted in our research unit as well as in farmer’s field. A desirable character, i.e., long shank introduced in this breed helps them for faster movement to escape from predators in the backyard condition; the parents of Vanaraja are selected for higher general immunity [6]. Vanaraja, a dual purpose chicken, has become popular among the rural people of as one of the income generating activity, especially for the rural women [7]. Particularly, these backyard breed is resistant to some common poultry diseases also. However, scanty information [8,9] is available on nutritional requirements of native chickens or strains for sustainable low input rural poultry production. The nutrients which have an immuno-modulating effect include protein and energy [10]. The variable energy protein offered to the birds affect the immune response of birds. Vanaraja strain is gaining popularity among poor farmers in India because of low input cost of production. However, there is no systemic study on this strain for different levels of energy and protein under hot, humid environment.

The two essential components such as protein and energy cost about 90% of the total feed cost which should be utilized most efficiently for desired economy of production and formulation of poultry ration. The protein and energy requirements of these birds are, however, not known. So, keeping in view, the present study was undertaken to investigate the effect of different levels of protein and energy sources on growth performance, immune status, and economics of Vanaraja chicks production during 1-56 days of age.

## Materials and Methods

### Ethical approval

The study was conducted following approved guidelines of the Institutional Animal Ethics Committee.

### Experimental design, management, and laboratory analysis

The experiment was conducted for 56 days with a view to investigate the influence of various level of energy and protein on the performance, immune response, and production economics of Vanaraja birds at the Poultry Nutrition Research Unit of Animal Nutrition Department, Bihar Veterinary College, Patna, India. Feed ingredients were procured in one lot for the whole experiment, and its proximate principles were determined as per AOAC [11] along with calcium and phosphorus using the method modified by Talapatra *et al*. [12] before compounding experimental rations, and feed formulation was done as per BIS [13]. Different ingredients used in the experiment were yellow maize, soybean meal, wheat bran, de-oiled rice bran, soybean oil, common salt, calcite powder, mineral mixture, and additives (Tables-1 and 2).

**Table-1:** Nutrient content of experimental diet (%, on DM basis).

Ingredients	DM	CP	EE	CF	TA	AIA	NFE	Ca	P	ME (kcal/kg)
Yellow maize	91.2	9.50	3.35	2.08	2.80	0.20	82.27	0.08	0.36	3330.18
Soybean meal	92.1	45.0	0.82	5.85	7.05	1.03	41.28	0.23	0.58	2450.62
Wheat bran	89.5	14.0	3.60	11.50	6.60	1.40	64.30	0.21	1.18	2000.82
De-oiled rice bran	92.5	13.0	1.78	13.25	6.40	2.70	65.57	0.07	0.98	1800.51

DM=Dry matter, CP=Crude protein, EE=Ether extract, CF=Crude fiber, TA=Total ash, AIA=Acid insoluble ash, NFE=Nitrogen free extract, Ca=Calcium, P=Phosphorus, ME=Metabolizable energy

**Table-2:** Percentage composition of different experimental diets.

Ingredients (%)	T_1_	T_2_	T_3_	T_4_	T_5_	T_6_	T_7_	T_8_	T_9_
Yellow maize	50.50	60.00	67.00	48.00	59.00	68.00	46.00	54.00	61.00
Soybean meal	19.00	21.00	22.00	25.00	27.00	27.50	31.00	32.00	33.50
Wheat bran	13.50	7.50	3.00	11.00	5.00	0.00	10.50	5.00	0.00
De-oiled rice bran	13.50	7.50	3.00	12.50	5.00	0.00	9.00	5.00	0.00
Soybean oil	0.00	0.50	1.50	0.00	0.50	1.00	0.00	0.50	2.00
Common salt	0.30	0.30	0.30	0.30	0.30	0.30	0.30	0.30	0.30
Calcite	1.00	1.00	1.00	1.00	1.00	1.00	1.00	1.00	1.00
Mineral mixture	1.50	1.50	1.50	1.50	1.50	1.50	1.50	1.50	1.50
Premix	0.70	0.70	0.70	0.70	0.70	0.70	0.70	0.70	0.70
Analyzed value									
CP (%)	17.05	17.10	17.15	19.04	19.20	19.15	21.08	21.10	21.19
ME (kcal/kg)	2607	2815	3009	2624	2810	3019	2609	2814	3012
Ca (%)	1.20	1.21	1.22	1.21	1.23	1.11	1.21	1.21	1.20
P (%)	0.54	0.53	0.54	0.52	0.54	0.51	0.54	0.54	0.54

Mineral mixture composition: Vitamin A (700,000 IU), vitamin D3 (70,000 IU), vitamin E (250 mg), nicotinamide (1000 mg), cobalt (150 mg), copper (1200 mg), iodine (325 mg), iron (1500 mg), potassium (100 mg), magnesium (6000 mg), manganese (1500 mg), selenium (10 mg), sodium (5.9 mg), sulfur (0.72%), zinc (9600 mg), calcium (25.5%), and phosphorus (12.75%). CP=Crude protein, ME=Metabolizable energy, Ca=Calcium, P=Phosphorus

A total of 600 1-day-old chicks of Vanaraja strain were procured from PDP, Hyderabad during the early winter season and temperature was approximate 32°C. The crippled chicks and those with extreme body weights were discarded from the study. Finally, 540 1-day-old chicks were individually weighed and distributed into nine groups having 60 birds in each. Each group was further subdivided into triplicates having 20 birds in each. Nine different experimental rations were formulated with three levels of protein, *viz*., 17%, 19%, and 21%; each with three levels of energy (2600, 2800, and 3000 kcal metabolizable energy [ME]/kg), respectively. Group T_8_ serves as control fed with 21% protein and 2800 kcal energy as per PDP, Hyderabad given requirement. Feed consumption, live weight gain, body weight change, feed conversion ratio (FCR), and performance index were calculated based on the amount of feed consumed every week.

### Immune status

The hyperimmune serum against a vaccine strain virus is obtained from the department of veterinary microbiology. This serum was inactivated at 56°C and stored at 0°C. Cell culture adapted live invasive intermediate infectious bursal disease (IBD) virus vaccine (EID50) available in freeze-dried form (Venkateshwara Hatcheries Pvt. Ltd., Pune) was used. The vaccine was reconstituted in diluents supplied with vial and used within few hours after reconstitution. A commercially available F-strain vaccine (Venkateshwara Hatcheries Pvt. Ltd., Pune) was used for vaccination of chicken after proper reconstitution at 7^th^ days of age. F-strain virus was further used as antigen in hemagglutination (HA) test and HA inhibition (HI) test, and 1.0% suspension of chicken red blood cell in phosphate buffer saline was used for HA and HI tests. The blood was collected from wing vein with 5 ml sterilized syringe using 22 gauge needles at the end of 7^th^, 14^th^, 21^st^, and 28^th^ days post-IBD vaccination. From each bird, 1-2 ml of blood was drawn and immediately transferred to sterilized test tubes which were kept in a slanting position and the blood was allowed to clot. The serum samples were collected and inactivated at 56°C for 30 min and finally stored at −20°C until use. HI test was performed in perspex plate as per the method suggested [14]. Finally, economics of broiler production was calculated on the cost of feed per kg live weight gain.

### Statistical analysis

Data obtained were subjected to analysis completely randomized design with the simple analysis of variance technique [15] using Statistical Package for the Social Sciences [16]. Homogenous subsets were separated using Duncan’s multiple range test described by Duncan [17]. Differences among treatments were considered to be significant when p≤0.05.

## Results

In this study, different parameters such as feed intake, body weight gain, FCR, immune response, and production economics were observed, respectively.

### Feed intake

The effect of different dietary levels of protein and energy on feed intake at the weekly interval and 8^th^ week of age in Vanaraja chicken showed a significant (p<0.05) effect and gradual changes were observed in feed intake in every week among the different treatment groups (Table-3). Feed intake during the entire experimental period, ranging from 2872.04 to 3129.66 g, which was significantly influenced by dietary treatment and level of protein and energy in the diet. Vanaraja chicken reared on 19% crude protein (CP) with 3000 kcal ME/kg showed lower feed intake than the treatment group fed with 17% CP, either increasing energy level. However, there was a significant difference in feed intake by broiler, reared on T_1_, T_2_, T_3_, T_4_, T_6_, T_7_, T_9_ and control groups, respectively.

**Table-3:** Effect of different levels of energy and protein on an average feed intake (g/bird), average body weight (g), average body weight gain (g) and FCR at weekly interval and 8^th^ week in Vanaraja chicken.

Week	T1	T2	T3	T4	T5	T6	T7	T8	T9
Percentage CP		17.0			19.0			21.0	
ME (kcal/kg)	2600	2800	3000	2600	2800	3000	2600	2800	3000
Feed intake (g/bird)									
1^st^	94.74^c^±1.63	112.00^e^±2.00	94.40^c^±4.00	86.78^ab^±1.50	90.86^abc^±0.50	102.00^d^±2.00	93.00^bc^±1.00	97.50^cd^±1.50	86.00^a^±2.00
2^nd^	145.23^bc^±4.78	152.50^c^±2.50	154.00^c^±4.00	153.64^c^±2.00	149.00^bc^±1.00	145.00^bc^±1.00	142.00^ab^±2.00	133.00^a^±3.00	133.50^a^±1.50
3^rd^	277.28^de^±4.72	281.00^e^±1.00	271.00^cd^±5.00	251.40^b^±1.00	246.00^b^±2.00	265.00^c^±1.00	234.50^a^±1.50	255.50^b^±3.50	246.00^b^±2.00
4^th^	390.22^e^±4.90	390.60^e^±5.00	370.84^d^±5.00	358.00^bc^±2.00	336.68^a^±2.00	342.00^a^±2.00	365.00^cd^±3.00	353.50^b^±1.50	382.50^e^±2.50
5^th^	460.06^ef^±7.94	452.90^de^±2.50	476.28^g^±5.00	438.00^c^±2.00	382.00^a^±2.00	406.50^b^±1.50	436.28^c^±2.00	446.00^cd^±2.00	466.66^fg^±2.50
6^th^	523.00^e^±5.00	511.00^cd^±1.00	503.74^bc^±3.50	501.50^b^±1.50	501.00^b^±1.00	473.50^a^±2.50	517.50^de^±1.50	513.50^d^±2.50	554.50^f^±2.50
7^th^	583.50^e^±7.50	578.00^de^±2.00	553.26^b^±2.50	526.50^a^±1.50	569.00^cd^±1.00	646.00^g^±2.00	565.50^c^±2.50	584.50^e^±3.50	626.50^f^±1.50
8^th^	602.00^e^±2.00	590.00^bcd^±2.00	543.40^a^±5.00	595.40^cde^±3.00	597.50^de^±1.50	601.50^e^±1.50	586.00^bc^±2.00	582.00^b^±6.00	634.00^f^±2.00
18^th^	3076.0^e^±13.67	3068.00^e^±6.00	2966.92^d^±1.00	2911.22^b^±9.50	2872.04^a^±6.00	2981.50^d^±1.50	2939.78^c^±1.50	2965.50^d^±5.50	3129.66^f^±4.50
BW (g)									
1^st^	90.80^a^±0.85	93.60^ab^±0.85	95.20^b^±1.16	96.40^b^±0.95	105.48^d^±1.26	110.72^e^±1.18	100.12^c^±1.16	111.20^e^±1.31	113.04^e^±1.29
2^nd^	181.44^a^±1.63	188.56^b^±2.21	190.76^b^±1.74	197.48^c^±1.21	216.72^d^±2.10	220.16^de^±1.49	200.92^c^±1.54	226.60^f^±2.78	223.28^ef^±1.12
3^rd^	267.56^a^±1.60	279.36^b^±1.76	294.60^c^±3.05	314.00^d^±2.87	326.16^e^±3.03	348.20^fg^±2.25	322.80^e^±3.54	341.00^f^±3.11	349.88^g^±2.34
4^th^	358.16^a^±2.34	384.64^b^±2.51	403.80^c^±4.22	421.20^d^±3.89	438.40^e^±3.20	487.40^g^±4.18	436.80^e^±5.47	463.20^f^±2.63	500.00^h^±5.57
5^th^	511.44^a^±4.83	565.80^b^±4.55	565.04^b^±1.73	572.60^b^±3.59	603.76^c^±4.56	645.40^e^±2.34	575.40^b^±3.39	631.92^d^±4.64	664.00^f^±5.59
6^th^	685.80^a^±5.97	738.32^b^±3.40	747.60^b^±4.53	763.80^c^±3.32	804.60^d^±3.89	865.80^fg^±2.64	770.40^c^±2.75	840.16^e^±5.90	900.40^g^±6.26
7^th^	864.80^a^±8.23	925.80^b^±2.67	936.00^b^±5.45	978.60^c^±5.93	1059.88^d^±6.00	1139.20^f^±4.57	976.00^c^±6.38	1104.60^e^±4.02	1167.80^g^±6.00
8^th^	1036.40^a^±7.78	1065.80^b^±4.91	1109.00^c^±4.74	1149.60^d^±10.32	1246.40^e^±5.16	1394.40^g^±5.72	1147.20^d^±8.94	1302.40^f^±5.39	1403.60^g^±3.91
BWG (g)									
1^st^	54.28^a^±1.04	56.60^ab^±0.80	57.64^ab^±1.39	59.28^b^±1.04	67.96^d^±1.35	72.72^e^±1.29	62.76^c^±1.25	72.92^e^±1.40	74.60^e^±1.22
2^nd^	90.64^a^±1.94	94.96^a^±2.39	95.56^a^±1.81	101.08^b^±1.47	111.24^cd^±1.82	109.44^c^±1.23	100.80^b^±1.94	115.40^d^±2.42	110.24^cd^±1.44
3^rd^	86.12^a^±2.20	90.80^a^±2.87	103.84^b^±3.19	116.52^cd^±2.66	109.44^bc^±2.58	128.04^e^±2.10	121.88^de^±3.55	114.40^cd^±2.60	126.60^e^±2.19
4^th^	90.60^a^±2.63	105.28^b^±3.21	109.20^b^±3.59	107.20^b^±4.23	112.24^bc^±2.93	139.20^d^±3.67	114.00^bc^±3.92	122.20^c^±3.03	150.12^e^±5.37
5^th^	153.28^b^±5.04	181.16^d^±4.11	161.24^bc^±4.12	151.40^ab^±5.55	165.36^bc^±5.50	158.00^bc^±3.33	138.60^a^±6.71	168.72^cd^±3.25	164.00^bc^±4.13
6^th^	174.36^a^±6.07	172.52^a^±5.02	182.56^ab^±4.02	191.20^bc^±4.89	200.84^cd^±5.12	220.40^e^±2.91	195.00^bcd^±4.45	208.24^de^±4.19	236.40^f^±6.34
7^th^	179.00^a^±8.46	187.48^ab^±4.14	188.40^ab^±7.70	214.80^c^±7.14	255.28^d^±4.91	273.40^d^±3.66	205.60^bc^±6.96	264.44^d^±6.98	267.40^d^±3.60
8^th^	171.60^b^±10.07	140.00^a^±5.14	173.00^b^±7.99	171.00^b^±10.09	186.52^bc^±5.23	255.20^d^±4.63	171.20^b^±11.62	197.80^c^±5.22	235.80^d^±7.35
18^th^	999.88^a^±7.84	1028.80^b^±4.95	1071.44^c^±4.67	1112.48^d^±10.27	1208.88^e^±5.18	1356.40^g^±5.68	1109.84^d^±8.94	1264.12^f^±5.31	1365.16^g^±3.84
FCR									
1^st^	1.44^e^±0.08	1.42^e^±0.12	1.37^d^±0.11	1.38^d^±0.15	1.26^b^±0.21	1.20^a^±0.13	1.31^c^±0.17	1.20^a^±0.22	1.18^a^±0.12
2^nd^	1.55^e^±0.15	1.51^d^±0.22	1.49^d^±0.12	1.43^c^±0.16	1.33^b^±0.24	1.29^a^±0.18	1.42^c^±0.23	1.27^a^±0.19	1.28^a^±0.13
3^rd^	1.90^h^±0.05	1.83^g^±0.09	1.78^f^±0.14	1.65^e^±0.11	1.55^c^±0.09	1.46^a^±0.15	1.60^d^±0.19	1.51^b^±0.12	1.46^a^±0.08
4^th^	2.26^g^±0.09	2.14^f^±0.19	2.08^e^±0.12	1.92^d^±0.18	1.78^b^±0.17	1.67^a^±0.08	1.85^c^±0.11	1.78^b^±0.07	1.67^a^±0.16
5^th^	2.52^f^±0.16	2.30^e^±0.13	2.31^e^±0.17	2.15^d^±0.06	1.94^b^±0.11	1.88^a^±0.15	2.08^c^±0.12	1.96^b^±0.09	1.86^a^±0.14
6^th^	2.71^f^±0.18	2.46^e^±0.21	2.48^e^±0.13	2.31^d^±0.16	2.09^b^±0.08	2.04^a^±0.13	2.25^c^±0.11	2.10^b^±0.12	2.04^a^±0.09
7^th^	2.85^g^±0.13	2.57^f^±0.07	2.60^e^±0.12	2.42^d^±0.19	2.21^b^±0.11	2.14^a^±0.23	2.38^c^±0.20	2.22^b^±0.18	2.13^a^±0.16
8^th^	2.95^e^±0.18	2.71^d^±0.11	2.70^d^±0.16	2.52^c^±0.08	2.31^b^±0.12	2.21^a^±0.14	2.50^c^±0.12	2.31^b^±0.14	2.22^a^±0.21
18^th^	2.96^e^±0.08	2.71^d^±0.21	2.71^d^±0.14	2.52^c^±0.13	2.31^b^±0.22	2.22^a^±0.20	2.51^c^±0.15	2.31^b^±0.11	2.22^a^±0.18

**abcdefgh:** Values with different superscripts in a row differ significantly (p<0.05). BW=Body weight, BWG=Body weight gain, FCR=Feed conversion ratio

### Body weight, body weight gain, and FCR

Result of body weight, body weight gain, and FCR at the weekly interval is presented in Table-3. The analysis of variance to saw the effect of different treatments on body weight and body weight gain in birds was found to be highly significant (p<0.05). A similar trend continued until the end of this experiment, where it was found that higher protein and energy level has a positive effect on body weight. The overall body weight gain in T_9_ group fed diet containing 21% CP, 3000 kcal ME/kg found to be highest, but it was significantly similar to T_6_ group, i.e., 19% CP and 3000 kcal ME.

During the entire experimental period, the FCR was significantly influenced by dietary treatment and level of protein and energy. It was observed that the FCR value is highest in T_1_ group 2.96 and significantly greater than other treatment group diet having 17% protein and 2800 kcal energy and T_2_ has comparable FCR with T_3_. Similarly, FCR value of T_4_, T_7_ and T_4_, T_8_ were not significantly different (p>0.05). The level of protein influenced FCR value, and the value was found to be significantly lower in 19% protein diet than 17% protein containing diet.

### Immune status

For assessment of the immune status of Vanaraja chicken, serum samples were subjected to HI test at 7, 14, 21, and 28 days post-IBD vaccination (day 14). HI titer to New castle disease (NCD) virus vaccine (F-strain) due to dietary protein energy variation was given in Table-4. The antibody titer for all observation ranged between 0.5 log_2_ and 5.0 log_2_. During the entire experimental period, it was inferred that the antibody titer of T_6_ group fed 19% CP, 3000 kcal ME/kg containing diet was comparatively highest. However, no significant (p>0.05) difference was found between T_9_ and control group. There was a synchronous increase in antibody titer against NCD virus as the level of protein and energy increase.

**Table-4:** Immune response against NCD virus vaccine (F-strain) due to dietary proteinenergy variation (log_2_).

Days post-IBD vaccination	HI titer (log_2_) of serum of experimental birds								

	T_1_	T_2_	T_3_	T_4_	T_5_	T_6_	T_7_	T_8_	T_9_
7^th^ day	0.00	0.00	0.00	0.00	0.00	0.00	0.00	0.00	0.00
14^th^ day	1.50^b^±0.34	0.67±0.21	0.83^b^±0.17	0.83^b^±0.17	0.50±0.22	0.83^b^±0.17	0.67±0.21	0.50±0.22	0.67±0.21
21^st^ day	2.00^b^±0.26	1.67±0.21	2.67^bc^±0.21	2.67^bc^±0.21	2.83^cd^±0.17	3.50^d^±0.22	3.33^cd^±0.21	3.33^cd^±0.21	3.17^cd^±0.31
28^th^ day	3.33±0.33	2.83±0.17	4.33^b^±0.21	4.33^b^±0.21	4.50^b^±0.22	5.00^b^±0.26	4.67^b^±0.21	4.67^b^±0.21	4.83^b^±0.17

abcd Values with different superscripts in a row differ significantly (p<0.05). NCD=New castle disease

### Economics of production

Economics as influenced by different levels of protein and energy is shown in Table-5. Total input cost per bird was calculated on the basis of total feed cost and cost of chicks, medicines, and other miscellaneous. As the level of protein and energy increases in diet increased, the cost of experimental ration also increases. However, when the cost of feed per kg live weight gain considered, it was found maximum in the T_6_ group fed diet containing 19% CP and 3000 kcal ME/kg and minimum in T_1_ group fed with 17% CP and 2600 kcal ME. Net profit per bird was also found highest in T_6_ and lowest in the T_1_ group. Result of economics also indicated that the profit margin was found to be more on the ration containing 19% CP 3000 kcal ME/kg than other dietary protein energy levels.

**Table-5:** Economics of production influenced by different dietary treatments in Vanaraja chicken rearing.

Attributes	T_1_	T_2_	T_3_	T_4_	T_5_	T_6_	T_7_	T_8_	T_9_
Feed cost/kg ration (Rs.)	18.00	18.50	19.00	20.00	21.00	22.20	23.40	25.00	23.50
Cost of ration consumed (Rs.)	30.80	32.10	34.15	36.50	37.0	38.50	39.60	44.10	41.10
Total feed cost (Rs.)	30.80	32.10	34.15	36.50	37.0	38.50	39.60	44.10	41.10
Cost of chicks+medicines+miscellaneous (Rs.)	43.50	43.50	43.50	43.50	43.50	43.50	43.50	43.50	43.50
Total cost (Rs.)	74.30	75.60	77.65	80.00	80.50	82.00	83.10	87.60	84.60
Average live weight of bird (kg.)	0.999	1.028	1.071	1.112	1.028	1.356	1.109	1.364	1.265
Market price of bird (Rs.) at the rate of Rs. 100/	99.90	102.8	107.1	111.2	120.8	135.6	110.9	136.4	126.5
Net profit/bird (Rs.)	25.60	27.20	29.45	31.20	40.30	53.60	27.80	48.80	41.90
Profit/kg live weight (Rs.)	25.85	26.45	27.49	28.05	33.36	39.52	25.06	35.7	33.12

## Discussion

The above results indicated that chicken reared on a higher level of protein and energy consumed less feed than the diet having lower level protein, which affects the feed intake than the level of energy. The present result agreed well with the finding of Sheriff *et al*. [18], who also obtained lower feed consumption in broiler fed 22% and 2670 kcal ME/kg ration containing a low level of CP and ME showed higher feed intake. Result of feed intake obtained in this study also corroborates the finding of Farrell *et al*. [19], who concluded that the feed intake was inversely related to energy concentration in the diet. The variation in feed intake could be due to energy content that associated with increase dietary energy concentration. Quail feeding with different levels of energy and protein did not affect feed intake of offspring [20]. A significant reduction in daily feed intake in chicks; when the energy concentration of the ration was increased by incorporating high fat contain maggot meal at the level of 8% in the diet [21]. This result is an agreement with observation [22] that feed efficiency improved and feed intake reduced with increasing dietary concentrations of these nutrients. Thus, a proper calorie protein ratio is needed in the ration for optimum intake of nutrient through feed consumption.

The body weight gain indicated that ration containing 19% and 21% CP at higher energy, gained maximum growth. The lower level of protein and energy was found to be poor performance on body weight gain. The results are in agreement with Verma and Pal [23] findings as high energy, and high protein had a positive effect on growth rate and was also reported by Bamgbose [21]. However, chicks fed diet with 19% and 21% CP with 3000 kcal ME/kg utilized feed more efficiently than the lower level of protein and energy in the diet. As reported by Haunshi *et al*. [24] that different ME levels had a significant effect on body weight gain, feed intake, and FCR. However, feeding the 19.64% CP diet was adequate, above which no significance improvement in performance was achieved in broiler chicken [25,26]. It is possible to reduce dietary CP level up to 10% after the starter period without any detrimental impact on growth performance [27]. Banerjee *et al*. [28] investigate the effect of feeding different levels of CP but similar levels of energy on Koekoeck chickens (dual purpose breed) and found that increasing in the level of protein in the diets did not influence the overall body weight gain and final live weight of chickens. However, the FCR improved numerically with increasing levels of protein in the diet. Thus, the present study showed better growth performance in Vanaraja chicken at 19% CP with lesser feed cost as compared to 21% protein containing diet [29].

As reported by Golian *et al*. [30] did not find any significant change in antibody titer due to the feeding of low energy diet. He further observed that high protein energy diet cause rapid growth and consequently decline immune response. The result was contradictory to the present finding. However, Enting *et al*. [31] showed that there was an increase immune response depending on the breeder age and egg weight. It is speculated that the better body weight gain corroborate health and antibody titer. However, Dahlem red laying hens required 2795 kcal/kg ME, 16% CP, 0.8% lysine, and 0.4% methionine for eliciting optimum performance and immune response during 28-40 weeks of age [32]. However, there was no significant effect on antibody titer against NCD vaccine [24]. Moreover, the better immune response recorded in the study might be due to better nutrient utilization and its extension toward the better immune response [33].

Previous work related to economics production with energy protein interaction on Vanaraja chicken was not reported. The best economical efficiency was recorded by quail chicks fed 20% CP with 1.05% lysine up to 42 days of age [34]. In contrast to the present result, Rao *et al*. [35] attain more profit margins in a ration containing 16% CP. The present study showed that the maximum profit attained with 19% CP diet with respect of the overall performance of Vanaraja birds rearing.

## Conclusion

It has been observed that there was positive inclination toward ration containing high protein and energy which also influence the immune response of Vanaraja chicken. To obtained desirable performance economically, ration containing 19% CP with 3000 kcal ME/kg diet should be adopted for Vanaraja chicken under farm condition.

## Authors’ Contributions

SP: Performed all work as a part of her thesis dissertation program. KK: Design of the experiment, technical help in executing the research, data analysis, writing, and correction of manuscript. C: Technical help and correction of manuscript. SK: Correction of manuscript. PKS: Data analysis and correction of manuscript. MK: Helps in doing immunological parameters and correction of manuscript. AD: Technical help and data analysis. All the authors read and approved the final manuscript.
